# *Trichomonas vaginalis* Transports Virulent* Mycoplasma hominis* and Transmits the Infection to Human Cells after Metronidazole Treatment: A Potential Role in Bacterial Invasion of Fetal Membranes and Amniotic Fluid

**DOI:** 10.1155/2018/5037181

**Published:** 2018-08-02

**Authors:** Tran Thi Trung Thu, Valentina Margarita, Anna Rita Cocco, Alessandra Marongiu, Daniele Dessì, Paola Rappelli, Pier Luigi Fiori

**Affiliations:** ^1^Department of Biochemistry, Hue University of Medicine and Pharmacy, Vietnam; ^2^Department of Biomedical Sciences, University of Sassari, Italy

## Abstract

*Mycoplasma hominis* is considered an opportunistic pathogen able to colonize the lower urogenital tract; in females the infection is associated with severe pregnancy and postpartum complications, including abortion, endometritis, preterm delivery, and low birth weight. Molecular mechanisms of pathogenicity and virulence effectors remain poorly characterized. A number of studies in the last decade have demonstrated that* M. hominis *can establish an endosymbiotic relationship with* Trichomonas vaginalis*, a urogenital parasitic protozoon, also associated with adverse pregnancy outcomes. Recently, two bacterial genes (*alr* and* goiB*) associated with amniotic cavity invasion and a single gene (*goiC*) associated with intra-amniotic infections and high risk of preterm delivery have been identified in* M. hominis *isolated from a group of pregnant patients. In this work we demonstrate that a high number of* M. hominis* intracellularly associated with* T. vaginalis* have* goiC* gene, in association with* alr* and* goiB*. In addition, we demonstrate that metronidazole treatment of* M. hominis*-infected* T. vaginalis* allows delivering viable intracellular* goiC *positive* M. hominis* from antibiotic-killed protozoa and that free* M. hominis* can infect human cell cultures. Results suggest that molecular diagnostic strategies to identify both pathogens and their virulence genes should be adopted to prevent severe complications during pregnancy.

## 1. Introduction

Preterm birth is a major cause of neonatal diseases and accounts for 75% of perinatal mortality [1, 2]. Preterm labor and premature rupture of membranes can be initiated by multiple mechanisms, but in most cases a precise cause cannot be established. In the last years, several studies have shown a significant and strong association between preterm birth and intrauterine infections, accounting for at least 25-40% of cases [3, 4]. Infections induce a robust inflammatory response that can stimulate uterine contractility and trigger spontaneous preterm labor [5, 6]. In most cases microorganisms reach the uterus and placental membranes via ascending route from vagina or through haematogenous spread from different sources [7].

*Mycoplasma hominis *is one of the microorganisms most commonly associated with preterm labor and it has been isolated in 40% of amniotic fluids showing infection [8].

Recent studies have suggested the occurrence of genetic variations among different* M. hominis* isolates with regard to their potential to invade the amniotic fluid and membranes. Three genes (*alr, goiB,* and* goiC*) have been identified in* M. hominis* isolated from amniotic fluids and the placenta of women with preterm labor, but not in the reference strain PG21, isolated from human intestine [9]. The gene* alr* encodes for alanine racemase and is involved in the peptidoglycan synthesis, yet the function of this enzyme in Mycoplasma species is not clear.* goiB* (gene of interest B) has unknown functions and encodes a protein that aligns with an* Ureaplasma urealyticum* hypothetical protein (41% identity and 63% similarity), while* goiC* (gene of interest C) encodes a 55 kDa polypeptide that appears to be strictly specific for* M. hominis*. Among the three genes,* goiC* is significantly associated with amniotic infection and preterm labor and can thus be considered a virulence trait of the* M. hominis* strains able to infect the amniotic cavity and the placenta [9].

Interestingly,* M. hominis *can establish a symbiotic relationship with* T. vaginalis,* a sexually transmitted protozoon associated with adverse pregnancy outcomes [10].* M. hominis* has the ability to enter and survive in protozoan cytoplasm, where it multiplies in coordination with the eukaryotic host [11]. It has been demonstrated that the percentage of* T. vaginalis *infected by* Mycoplasma hominis *ranges from 5 to 89% regardless of the geographic origin [12–15]. For a recent review see Fichorova et al. [16]. The* M. hominis-T. vaginalis* consortium strongly influences the pathobiology of the protozoon and contributes to upregulating the host inflammatory response to the infection [17–19].

The protozoon* Trichomonas vaginalis *has been also linked to preterm birth, but in this case the infection is limited to the vagina, without reaching the uterus and placental membranes [20]. The effective role of* T. vaginalis *in preterm labor is debated, since the mechanisms involved are still unclear. Data on protective effect of metronidazole treatment on adverse pregnancy outcomes are contradictory. In some studies antibiotic therapy seems to be effective in preventing adverse pregnancy complications, but several papers report a failure to prevent preterm delivery by metronidazole treatment in pregnant women with* T. vaginalis *infection [21, 22].

The first objective of this work was to understand if* goiC *(in association with* alr *and* goiB)* is present in* M. hominis* able to establish intracellular symbiosis with* T. vaginalis* clinical isolates, thus representing an additional potential risk factor for adverse maternal outcomes during trichomoniasis. In addition, we set up an* in vitro* model to assess the transmissibility of virulent (i.e.,* goiC *positive)* M. hominis* released from metronidazole-treated* T. vaginalis* to human-derived cells. Results obtained suggest an active role for protozoa not only in transport but also in transmission of bacterial infection to human tissues during pregnancy.

## 2. Material and Methods

### 2.1. Cells and Culture Conditions

A total of 34* T. vaginalis* strains were isolated in Italy and Mozambique from 1994 to 2017. Protozoa were isolated from vaginal swabs of women with trichomoniasis by inoculation in Diamond's TYM medium (trypticase, yeast extract, maltose) supplemented with 20% fetal bovine serum (FBS), penicillin (300 U.I/ml), and streptomycin (300 mg/ml), in order to eliminate concomitant vaginal flora [23]. Protozoa were then cultured by 1:16 daily passages in Diamond's TYM medium without antibiotics at 37°C in a 5% carbon dioxide atmosphere for at least three weeks and stored at −80° until use.

The immortalized human cell line WISH (ECACC catalog code: 88102403) was maintained in RPMI 1640 supplemented with 10% FBS at 37°C in a 5% carbon dioxide atmosphere, in T-25 flasks. Once cell cultures reached the confluence (twice a week) adherent cellular monolayer was enzymatically dissociated with trypsin, and detached cells were passed, 1/10-1/20, in complete RPMI medium supplemented with 10% fetal bovine serum.

*M. hominis* isolated from* T. vaginalis *and the bacterial reference strain PG21 were cultivated in SP4 agar plates and SP4 broth.

Minimal Inhibitory Concentration (MIC) for metronidazole and gentamicin were calculated by incubating* T. vaginalis* SS-49 and* M. hominis* isolated from the same protozoan strain, with serial dilutions of drugs in liquid media (Diamond's TYM and SP4 broth, respectively).

### 2.2. Selection of* M. hominis*-Parasitized* T. vaginalis* Isolates

The presence of* M. hominis *in* T. vaginalis *in the 34 strains included in this study was assessed by specific PCR. For each strain, DNA was extracted from 10^6^ mid–log-phase trichomonad cells as previously described. DNA was then resuspended in TE buffer (10 mM Tris-HCl pH 8, 1 mM EDTA) at a final concentration of 0.1 *μ*g/*μ*l and subjected to a Multiplex PCR assay for the detection of* T. vaginalis *and* M. hominis* [24].

*M. hominis *were isolated from each positive* T. vaginalis *strain in selective media. Briefly, protozoan cultures were centrifuged at 350 x g, and* T. vaginalis*-free culture supernatants were filtered through a 0.45-*μ*m filter membrane and finally inoculated in SP4 agar plates. Plates were incubated at 37°C until the appearance of detectable fried egg shape colonies on the agar surface. Bacterial DNA was extracted as described.

### 2.3. Identification of Virulence Genes in* M. hominis* Associated* with T. vaginalis*

The presence of genes* alr, goiB*, and* goiC *in* Mycoplasma hominis* isolated from protozoan strains was assessed by specific PCR as described by Allen-Daniels et al. [9]. The intestinal* M. hominis* reference strain PG21, lacking the* alr, goiB*, and* goiC* genes, and the* T. vaginalis *reference strain G3 that is not parasitized by* M. hominis, *were used as negative controls. In order to confirm PCR results, all amplicons were sequenced (BMR Genomics, Padova, Italy).

### 2.4. Transmissibility of* M. hominis* Infection from Metronidazole-Treated* T. vaginalis* to Human-Derived Cells

The transmissibility of intracellular* M. hominis* from* Mycoplasma*-infected* T. vaginalis* to human-derived cells was studied. Exponentially growing* T. vaginalis* SS-49, naturally infected by* M. hominis* positive for* alr, goiB,* and* goiC* genes, were extensively washed in phosphate buffered saline (PBS), in order to eliminate nonadherent extracellular mycoplasma cells. Protozoa were then resuspended in 500 *μ*l of RPMI medium supplemented with 10% FBS, added to a T-25 flask of semiconfluent WISH cells at 2:1 protozoa/human cells ratio, and incubated at 37°C in a 5% CO_2_ atmosphere. After 30 minutes of incubation, cells were extensively washed with PBS to remove protozoa and WISH cells were trypsinized

The effect of metronidazole on* Mycoplasma* transmissibility was assessed in a second experiment: WISH cells, infected with* T. vaginalis* as described above for 30 minutes, were treated by adding to the flask 25 *μ*g/ml of metronidazole (ten times the minimal lethal concentration for the strain SS-49, data not shown). After 24 hours of incubation, cells were extensively washed in PBS to eliminate killed protozoa and cellular debris and trypsinized as described.

In order to demonstrate that* M. hominis* associated with* T. vaginalis *can invade human cells and survive intracellularly, the same experiment has been carried out adding a further step. Briefly, after 24 hours of incubation of WISH cells in the presence of* T. vaginalis* SS-49 with metronidazole, gentamicin was added to the cells at concentration of 50 *μ*g/ml (four times the minimal lethal concentration for the* M. hominis* strain, data not shown) for 3 hours to kill extracellular bacteria. Cultures were then extensively washed in PBS and cultured for three additional weeks in complete RPMI 1640 medium, without any drugs. In all experiment the number of* M. hominis* cells associated with WISH cells was quantified by qPCR according to the protocol described by Ferandon et al. [25].

In all experiments, one aliquot of trypsinized cells was inoculated into BE broth to reisolate* Mycoplasma hominis*, and a second aliquot was subjected to DNA extraction as previously mentioned. All the experiments were carried out in triplicate.

## 3. Results

### 3.1. *T. vaginalis* Isolates Can Be Parasitized by* goiC* Positive* M. hominis* Strains

**Figure 1:**
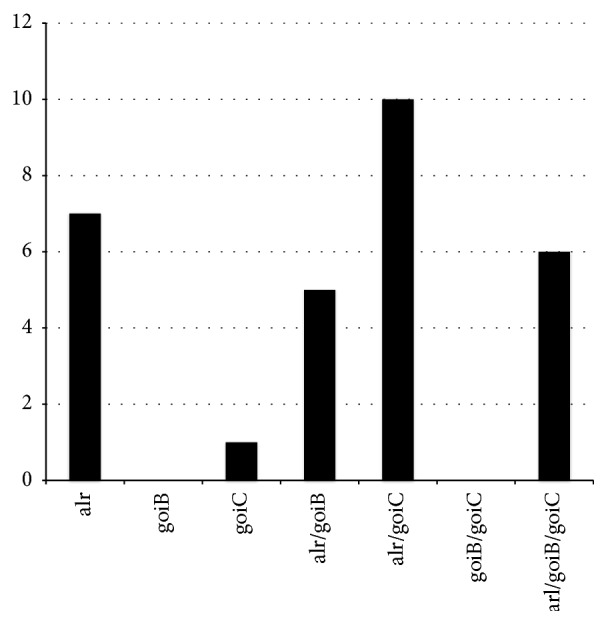
Association among* alr*,* goiB*, and* goiC* genes in a group of 29 samples of DNA extracted by T. vaginalis.

We demonstrated by PCR that 29 out 34* T. vaginalis* isolates included in this study were stably parasitized by* M. hominis*.* M. hominis* were isolated in SP4 medium from all* T. vaginalis* positive strains, and the presence of* goiC*,* arl,* and* goiB* virulence genes associated with adverse pregnancy outcomes and preterm delivery was assessed by PCR. Among 29* M. hominis* tested the* goiC* gene is present in 17 strains (58.2%), while* goiB* and* alr* are detected, respectively, in 11 (37.93%) and 28 (96.55%) isolates. The* goiC* gene is strongly associated with* alr* (16/17) and only partially with* goiB* (6/17). Only 6 out 29 (20.7%)* M. hominis* have all three genes.* M. hominis* reference strain PG21 and the* T. vaginalis* reference strain G3, that is* M. hominis *free, do not possess* alr, goiB*, and* goiC* genes. Results are shown in Figure 1.

### 3.2. *T. vaginalis* Parasitized by Virulent* M. hominis* Can Deliver Bacteria Able to Infect Human Cells

We hypothesize that the massive release of virulent intracellular* M. hominis* from metronidazole-killed* T. vaginalis* following antiprotozoan therapy could mediate the infection of amniotic fluid and membranes. In order to demonstrate this hypothesis, we set up in vitro experiments coculturing a human-derived cell line WISH and* T. vaginalis* infected with* M. hominis* positive for all virulence genes (*alr, goiB, *and* goiC*). First experiment was carried out coincubating protozoa and WISH cells for 30 minutes in absence of metronidazole. Results obtained by qPCR show that* M. hominis* delivered from Mycoplasma-parasitized* T. vaginalis* live cells can infect less than 0.2% of WISH cells after 30 minutes of incubation.

On the contrary,* M. hominis* were massively delivered from metronidazole-killed protozoa after 24 hours of incubation and viable bacteria were able to infect human cells with a multiplicity of infection (MOI) corresponding to 1.2* M. hominis*/3 WISH cells. These data suggest that free bacteria quickly adhere to human target cells once delivered from killed protozoa. In all experiments, infecting* M. hominis* were reisolated from mammalian cells by using mycoplasma-specific media.

A further experiment was assessed adding gentamicin in medium cultures (gentamicin protection assay), in order to establish if* M. hominis* released by* T. vaginalis* treated with metronidazole can also invade and chronically survive into human cells. qPCR demonstrate that* M. hominis *can survive intracellularly for three weeks in WISH cells exposed to gentamicin, with a MOI corresponding to 1 bacterium/4 human cells. Results were also confirmed by isolation of bacteria from gentamicin treated human cells in mycoplasma-specific media.

Results obtained suggest that* M. hominis* released by* T. vaginalis* after treatment with metronidazole can lead to chronic infection in fetal membranes and amniotic fluid, causing adverse pregnancy outcomes, and can also explain the pharmacological failure in preventing adverse pregnancy complications by use of metronidazole to treat subacute trichomoniasis.

## 4. Discussion

Preterm birth is a major cause of neonatal illness and death, especially in developing countries. Local and systemic microbial infections are important causes of preterm labor and premature rupture of membranes [1, 26].* M. hominis* and* T. vaginalis* infections are both associated with adverse pregnancy outcomes.* T. vaginalis *limits its colonization to the vagina [27, 28], and the infection seems to play a role in adverse pregnancy outcomes by inducing a massive local inflammation and the production of proinflammatory cytokines [29, 30]. On the contrary,* M. hominis* can invade the amniotic cavity, thus directly exploiting virulence mechanisms in this microenvironment. Even if mechanisms of pathogenicity and virulence genes involved in adverse pregnancy complications associated* M. hominis *are not fully characterized, Allen-Daniels and colleagues recently identified two genes (*arl* and* goiB*) in bacterial strains isolated in amniotic fluid and placental tissue and a third gene (*goiC) *that is significantly associated with amniotic fluid invasion and preterm labor risk [9]. Even if the effective function of the three genes is not clear, the authors hypothesize that* goiC* could contribute to the colonization of the placenta and the amniotic fluid rather than the vagina [9].

*M. hominis *infection can be mediated* in vivo *by* T. vaginalis, *since the two microorganisms are able to establish a symbiotic relationship, and most protozoan isolates are stably infected by the bacterium [10]. In a study conducted in Italy, 78.6% of women with trichomoniasis were affected also by* M. hominis *[24]. So far, the presence of* alr, goiB, *and* goiC *genes has been verified only in* M. hominis* isolated from patients that had no history of trichomoniasis. We investigated the presence of* alr, goiB, *and* goiC *genes in* M. hominis* strains that live in symbiosis with* T. vaginalis. *Our data reveal that not only free* M. hominis* but also those that live in symbiosis with* T. vaginalis* can possess the three genes associated with amniotic membranes colonization and adverse pregnancy outcomes.

The ability of* M. hominis* isolates to locate intracellularly has been previously demonstrated by several authors, in different human-derived cell lines and spermatozoa [31–34]. Hopfe et al. demonstrated that mycoplasmal infection of host cells is mediated by bacterial cytoadhesins [31]. In addition, Henrich et. al characterized several* M. hominis* genes involved in HeLa cells intracellular infection [35].

We demonstrate that* M. hominis *released by* T. vaginalis *are able to infect WISH cells* in vitro*. However, since* T. vaginalis* is highly cytopathic and induces a massive destruction of the cell monolayer in less than 2 hours, we had to limit the coincubation to a very short time. To prevent target cell lysis, we added metronidazole to the cells after 30 minutes of coincubation, demonstrating that* M. hominis* released by* T. vaginalis *and killed by the drug can efficiently and stably invade human cells. The quantification by qPCR of* M. hominis* associated (i.e., membrane associated and intracellular bacteria) with human cells after 24 hours of coincubation of metronidazole-treated* T. vaginalis* and WISH cells reveals that about 40% of mammalian cells are infected by the bacteria.

Results obtained by gentamicin protection assays demonstrate the ability of* M. hominis* released by metronidazole-killed* T. vaginalis* to locate intracellularly in mammalian cells. In fact, since gentamicin kills only the extracellular* M. hominis,* the detection of bacteria in infected WISH after three weeks of cultivation in the presence of gentamicin indicates that human cells can be chronically infected by intracellular bacteria, as previously demonstrated by Hopfe by using HeLa cells [31]. These data confirm the ability of mycoplasmas released by* T. vaginalis *to infect human host cells and to locate intracellularly, suggesting a role of* T. vaginalis *infection in transmission of* M. hominis*. A similar result has been reported by Fichorova et al.: in an elegant paper the authors demonstrated that metronidazole-killed* T. vaginalis* can deliver intracellular endobiont dsRNA virus (TVV) and that free viruses, even if they are unable to directly infect human cells, can stimulate a massive proinflammatory response: the production of cytotoxic cytokines can lead to severe complications during pregnancy [36].

These results suggest that the role of protozoan infection in adverse pregnancy outcome could not be limited to the induction of vaginal inflammation. In fact, the peculiar* T. vaginalis/M. hominis* symbiosis represents an additional potential risk factor for adverse maternal outcomes and preterm delivery during trichomoniasis.* T. vaginalis* can carry* M. hominis* possessing the* alr, goiB,* and* goiC* genes, protecting them intracellularly from the host immune response and antibacterial therapy, thus allowing their multiplication and transmission. The intracellular localization of bacteria in* T. vaginalis *cells can explain the paradoxical results described by some authors, reporting the failure of metronidazole treatment of subclinical trichomoniasis to prevent preterm delivery in pregnant women [21, 22]. In this scenario, the anti-*T. vaginalis* treatment with drugs selectively effective against trichomoniasis, probably together with cytolysis of protozoa mediated by host immune response, could induce a massive release of* M. hominis* from killed* T. vaginalis*, leading to bacterial invasion of placental membranes and amniotic fluid.

## 5. Conclusions

The symbiotic consortium between* T. vaginalis *and* M. hominis* implies a role in infections during pregnancy: we can hypothesize a primary role for* T. vaginalis *as “Trojan horse”, able to transport virulent bacteria, protecting them not only from local massive host innate and adaptive immune response, but also from antimycoplasma antibiotics unable to cross the protozoan membrane. In consequence, molecular diagnostic strategies to identify both pathogens and their virulence genes might be adopted to prevent severe complications during pregnancy.

## Data Availability

The data used to support the findings of this study are available from the corresponding author upon request.
